# Plasma fluorination of vertically aligned carbon nanotubes: functionalization and thermal stability

**DOI:** 10.3762/bjnano.6.232

**Published:** 2015-12-01

**Authors:** Claudia Struzzi, Mattia Scardamaglia, Axel Hemberg, Luca Petaccia, Jean-François Colomer, Rony Snyders, Carla Bittencourt

**Affiliations:** 1 Chimie des Interactions Plasma-Surface, CIRMAP, University of Mons, 7000 Mons, Belgium; 2 Materia Nova Research Center, 7000 Mons, Belgium; 3 Elettra Sincrotrone Trieste, Strada Statale 14 km 163.5, 34149 Trieste, Italy,; 4 Research Group on Carbon Nanostructures (CARBONNAGe), University of Namur, 5000 Namur, Belgium

**Keywords:** carbon nanotubes, spectroscopy, synchrotron radiation, thermal stability

## Abstract

Grafting of fluorine species on carbon nanostructures has attracted interest due to the effective modification of physical and chemical properties of the starting materials. Various techniques have been employed to achieve a controlled fluorination yield; however, the effect of contaminants is rarely discussed, although they are often present. In the present work, the fluorination of vertically aligned multiwalled carbon nanotubes was performed using plasma treatment in a magnetron sputtering chamber with fluorine diluted in an argon atmosphere with an Ar/F_2_ ratio of 95:5. The effect of heavily diluted fluorine in the precursor gas mixture is investigated by evaluating the modifications in the nanotube structure and the electronic properties upon plasma treatment. The existence of oxygen-based grafted species is associated with background oxygen species present in the plasma chamber in addition to fluorine. The thermal stability and desorption process of the fluorine species grafted on the carbon nanotubes during the fluorine plasma treatment were evaluated by combining different spectroscopic techniques.

## Introduction

The covalent functionalization of carbon nanostructures has been largely exploited, and different techniques have been employed for achieving fine control of their electronic properties. Carbon nanostructures have been decorated with a large variety of atoms and molecules, using wet chemistry, hydrothermal reactions and plasma process [1–6]. Among the most studied, fluorine-based grafting species represent both a valid precursor for several reactions (the introduction of polar groups has been successfully adopted to initiate subsequent functionalization [7–10]) and a solution for profitably implementing the carbon-based nanomaterials in several applications, such as gas sensors, batteries and polymeric switches [11–15]. A key characteristic of fluorine-based species is the different chemical interactions in carbon–fluorine bond formation [16–17]. Primarily covalent bonding occurs as a result of the plasma process, which is easily verified by different techniques such as Fourier transform infrared spectroscopy (FTIR), Raman spectroscopy and X-ray photoelectron spectroscopy (XPS) [10,18–19]. Although a large number of fluorination strategies and characterization routes have been reported, the choice of the precursor gas is a crucial issue that should not be underestimated due to the risk of polymerization, the introduction of unwanted atoms on the functionalized system, or the high toxicity level of the gas used. Carbon tetrafluoride (CF_4_), sulfur hexafluoride (SF_6_), pure fluorine (F_2_) or diluted fluorine in an inert atmosphere (Xe/F_2_, Ar/F_2_) are the precursor gases most commonly used. In the case of fluorine and noble gases mixtures, an important role for achieving optimal fluorination is played by the relative concentration of fluorine in the mixture during the plasma activation.

The previous studies on vertically aligned carbon nanotubes (vCNTs) fluorinated by Ar/F_2_ plasma treatment allowed for the evaluation of the spatial distribution of fluorine atoms, while X-ray photoelectron spectromicroscopy measurements indicated that the grafting occurred mainly up to a few µm under the tips of the nanotubes without damaging the carbon structure [20]. In that case, the Ar/F_2_ mixture concentration used was a ratio of 90:10. The purpose of the present study is to discuss the impact of more diluted fluorine gas in an argon atmosphere (Ar/F_2_, 95:5) on the fluorination yield and to evaluate its effect in the fluorination mechanism of vCNTs, aiming at controlled fluorine grafting at the tip of the vCNTs. A temperature-dependent study was performed and the resulting defluorination process is discussed based on the analysis carried out using different techniques such as XPS, ultraviolet photoemission spectroscopy (UPS) and Raman spectroscopy. We observed that oxygen present in the plasma chamber, mainly as water vapor, is also grafted on the CNTs surface in addition to fluorine species leading to oxyfluorination of the vCNTs. The fluorine functionalization causes the hybridization change from sp^2^ to sp^3^ of the carbon atoms. We show that controlled thermal heating of the sample allows for a fine selection of grafted species and tuning of electronic properties.

## Experimental

vCNTs were produced by catalytic chemical vapor deposition (CCVD) at atmospheric pressure. The catalysts were prepared by magnetron sputtering; first, a 30 nm Al_2_O_3_ buffer layer was deposited on Si wafers with native SiO_2_, next, a 6 nm Fe layer was then added on top of the Al_2_O_3_ buffer layer to form (after annealing) nanoparticles which catalyze the nanotube growth. For the vCNTs growth, the catalyst was placed inside the reactor, heated to 750 °C at atmospheric pressure under Ar flow (120 sccm), then an additional flow of hydrogen (120 sccm) was introduced. After 5 min, Ar was replaced by ethylene (C_2_H_4_) flow (50 sccm) for 20 min. After the growth, the oven atmosphere was again filled with Ar.

Fluorination was performed by exposing the vCNTs samples to fluorine chemical species generated in a magnetron sputtering chamber with base pressure of about 5·10^−6^ mbar, using a graphite target. The plasma discharge of F_2_ (diluted in Ar in a ratio 95:5) was produced by an ENI RPG 5 kW asymetric bipolar pulsed DC power supply with a period of 4 µs and a pulse duration of 1.6 µs. The following functionalization parameters were used: mean power, *P* = 100 W; gas flux, Φ = 10 sccm; working pressure, *p*_w_ = 30 mTorr; functionalization time, *t* = 900 s.

The chemical modifications due to fluorine grafting were evaluated by XPS and UPS. The experimental geometry of the data collection allowed for the analysis of the tip of the vCNTs – this region of the sample is referred to as the surface. The chemical composition was studied using a VERSAPROBE PHI 5000 from Physical Electronics, equipped with a monochromatic Al Kα X-ray source. The energy resolution was 0.6 eV. For the compensation of built-up charge on the sample surface during the measurements, a dual beam charge neutralization composed of an electron gun (≈1 eV) and an Ar ion gun (≤10 eV) was used. The valence band investigation was performed using an excitation photon energy of *h*ν = 31 eV from a horizontally polarized (p-polarized) synchrotron light source at the BaDElPh beamline of the Elettra synchrotron in Trieste, Italy [21]. A temperature-dependent study was performed by thermal heating in ultrahigh vacuum: the selected temperature was reached in about 20 min, and the sample was kept for 15 min at that temperature before turning off the heating. The structural changes as a result of functionalization were evaluated by Raman spectroscopy. The Raman spectra were collected using a Senterra Bruker micro-Raman system spectrometer with a laser wavelength of 532 nm as the excitation source. The micro-Raman system provides a spectral resolutions of 5 cm^−1^. The laser power impinging on the sample was kept constant at 2 mW to avoid heating and a 50× objective was used. Five measurements were acquired at different locations on the as-functionalized sample and after two heating steps (*T* = 540 °C and 900 °C) and averaged. The Raman peaks were fitted using Lorentzian functions after baseline correction. In all analyses, the spectra were acquired at room temperature (RT) once the sample recovered after heating in UHV conditions at the desired temperature.

## Results and Discussion

We first investigated the functionalization of vCNTs using XPS. In a pure fluorination process, only the fluorine signal is expected in addition to carbon; however, the oxygen signal was also observed for the as-functionalized sample, where [F] = 24 at % and [O] = 13 at % concentrations were found. The oxygen atoms present in the sample influence the chemical environment of the carbon atoms at the surface, acting as grafting species that are often present in the residual vacuum (in the form of water vapor and molecular oxygen) and are decomposed by the plasma process [22–23]. The Ar atoms in the gas mixture strongly affect the functionalization since the Ar ions are mainly responsible for creating defects on the carbon surface, that is, active sites for grafting fluorine and oxygen species [24–26].

**Figure 1:**
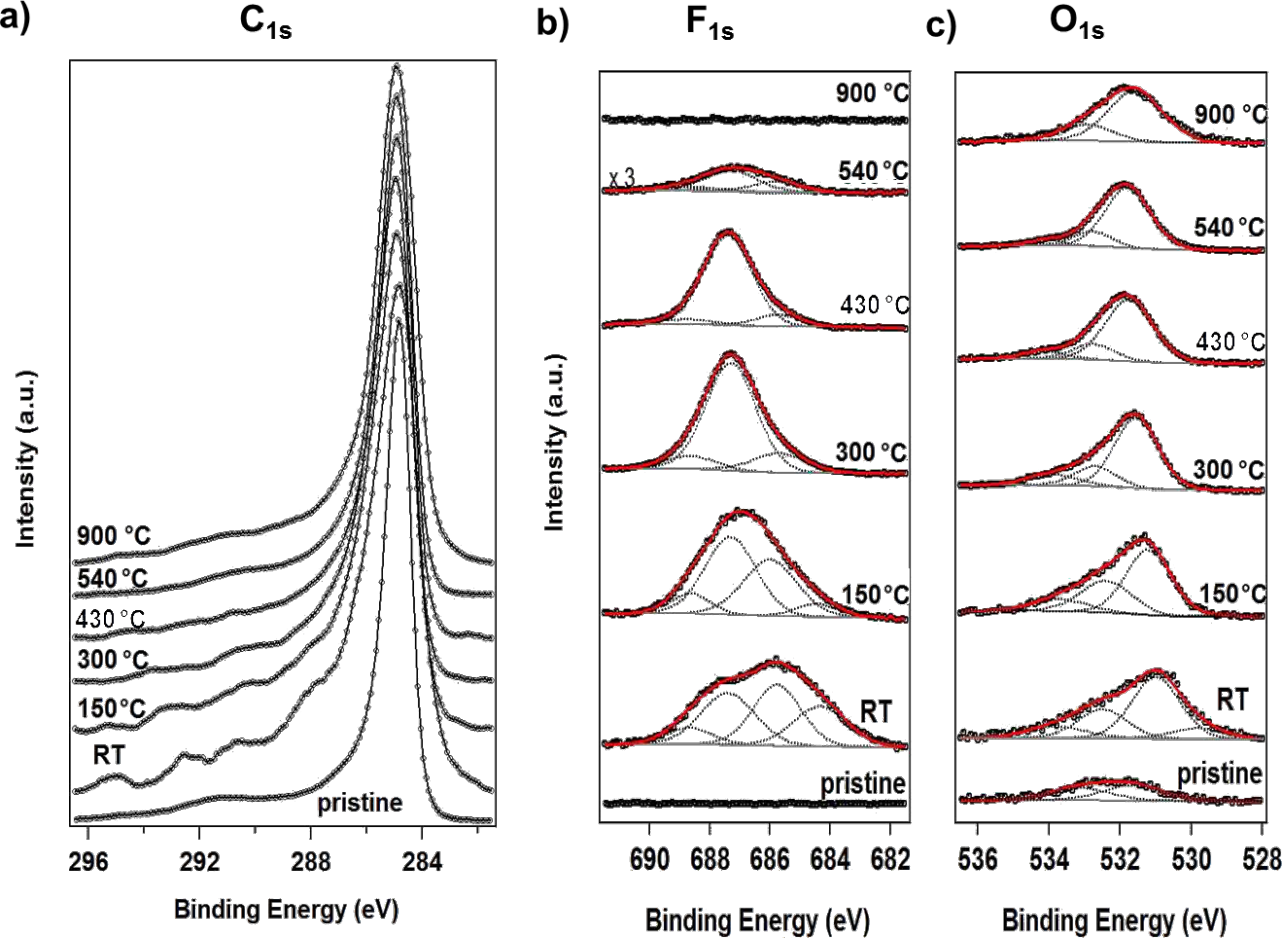
Temperature-dependent XPS analysis of functionalized vCNTs. (a) C 1s spectra acquired as a function of heating temperature. The C 1s spectrum recorded just after the fluorination is labelled RT, the pristine spectrum is the bottom curve. The spectra were normalized and stacked for better visualization of changes in their lineshape. (b,c) Fitting curves of the experimental data (black dots) acquired from F 1s and O 1s core levels as a function of heating temperature.

In Figure 1 we show the changes of the vCNTs chemical composition at the surface (tips) with increasing heating temperature. In order to evaluate the thermal stability of the grafted species, we first analyze the C 1s, F 1s and O 1s core levels in the XPS spectra acquired after fluorination (labeled RT in the figure). Once the Shirley-type background was subtracted, the C 1s intensities in Figure 1a were normalized to the maximum value, allowing a direct visualization of the plasma-induced modifications in the C 1s peak lineshape as a function of heating temperature. The F 1s and O 1s spectra presented in Figure 1b,c, respectively, show the fitting procedure used to reproduce the lineshape and changes in the fitting components upon heating (details on the fitting parameters are reported in Supporting Information File 1, Table S1).

The grafting of fluorine species on the carbon nanotube surface induces modifications in the C 1s lineshape (Figure 1a). We observe that, compared to the pristine spectrum, the spectrum recorded after fluorination shows new structures at binding energies higher than 285 eV. The deconvolution of the carbon peak is complex due to the uncertain assignment of fitting components; however, a satisfactory example is reported in Supporting Information File 1 for the as-functionalized sample (Supporting Information File 1, Figure S1). The analysis of the lineshape confirms the presence of C–C in sp^2^ (284.5 eV) and sp^3^ (285.1 eV) bonding configurations, C–O (hydroxyl 285.9 eV, carbonyl 287.0 eV, carboxyl 289.2 eV) [2,27–28] and C–F species, which can be classified into two main groups related to primary and secondary shifts of the carbon component. More specifically the following assignments can be made: carbon atoms indirectly linked to fluorine but first neighbors of C–F bonds (β-position), such as C–CF located at 286.2 eV, and carbon directly bound to fluorine atoms, such as covalent C–F around 288.0 eV, CF–CF*_n_* at 290.7 eV, CF_2_ at 292.5 eV and CF_3_ at about 295.0 eV [16,19,29–31]. The latter fluorinated carbon species are the first to desorb; therefore, they represent the less thermally stable species due to their instability when bound to edges as open tube ends. This can be caused by the impact of energetic ions in the Ar/F_2_ plasma with the vCNTs surface. During plasma treatment, partial sputtering of the carbon atoms from the tips can be produced, as confirmed by the component centered at 283.5 eV used to reproduce the C 1s peak. This accounts for the increased intensity at the low binding energy side of the C–C sp^2^ component. This low binding energy feature (labeled *C*_def_ in Supporting Information File 1, Figure S1) has been associated with ion-induced defective carbon [32–33]. It is important to mention that partial recovery of the pristine structure was obtained due to the self-healing ability of the hexagonal carbon lattice when the sample is thermally heated, as verified by the disappearance of this low binding energy component.

**Figure 2:**
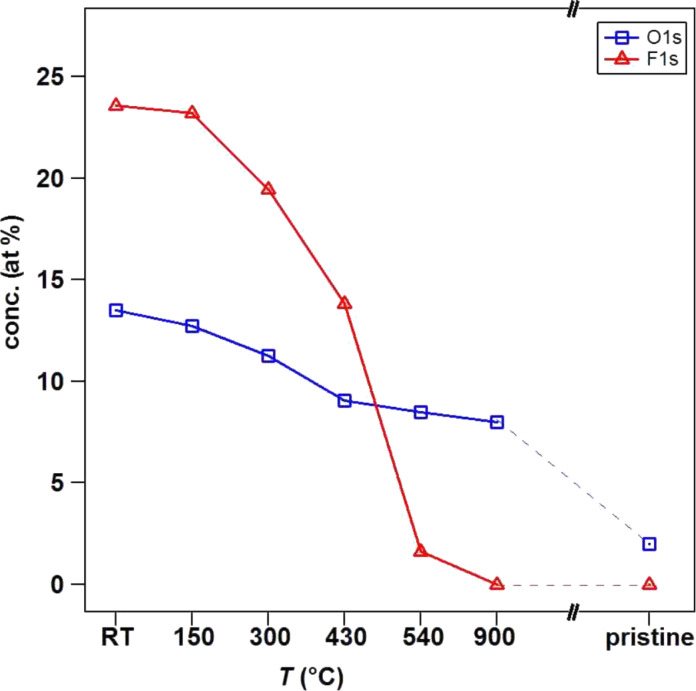
Atomic concentration of oxygen (blue squares) and fluorine (red triangles) calculated from XPS analysis as a function of heating temperature.

In Figure 1b,c we report the experimental data (black dots) related to F 1s and O 1s core level regions together with the fitting components used. The overall asymmetric lineshapes of F 1s and O 1s evidence the influence of their respective electronegativity in the electronic environment. Four components are used for the fitting of both spectra. In particular, at room temperature we can distinguish adsorbed fluorine (684.3 eV), “semi-ionic” C–F bonds (685.8 eV), covalent C–F bonds (687.4 eV) and a contribution from fluorine atoms bound to multiple fluorinated carbon (as CF_2_) mainly located at the edges and local defect sites (688.7 eV) [16,27,30,34–35]. For oxygen, however, the following contribution are found: O=C–OH (529.8 eV), C=O (531.0 eV), C–O (532.5 eV), and oxygen bound to fluorinated carbon or to C atoms located near fluorinated carbon atoms (533.8 eV) [30,34,36]. The latter is expected in the case of fluorination in an oxygen-containing atmosphere due to the grafting of oxygen species [37]. In parallel to the decrease in intensity of the components used to reproduce the C 1s peak, the degree of asymmetry of F 1s and O 1s decreased drastically upon heating. This suggests a different desorption temperature and hence distinct binding energies of the several species grafted on the carbon surface, as it will be discussed. After heating the sample to 150 °C, we observed that the intensity of the components in the C 1s spectrum related to multiple fluorinated carbon decreased, where a similar effect was observed in the F 1s core level spectrum for the component at 688.7 eV. This occurs concurrent with the desorption of adsorbed fluorine represented by the peak at 684.3 eV. The defluorination process is activated by increasing the temperature from 300 to 500 °C, and the component associated with the covalent C–F bond becomes the dominant feature with respect to the other components that show desorption at lower temperature. After heating at a higher temperature (*T* = 900 °C), only C–O contributions were observed, as confirmed by Figure 1b,c. Moreover, in the O 1s spectrum of the as-functionalized sample (RT), we observed a shift in the C–O and C=O components with respect to the pristine ones. This is likely related to different charge distributions in the atoms when the fluorine quantity is high, changing the chemical environment and consequently modifying the screening of the atoms. In parallel to desorption of fluorinated species, the oxygen components backshift towards the binding energies observed for the pristine sample, indicating the coexistence of grafted species in neighboring sites. This can be explained by preservation of the strongest covalent C–F bonds in conjunction with the vanishing of adsorbed fluorine present in the material and the reduction of the amount of “semi-ionic” C–F bonds, leading to an overall decrease of fluorine concentration. The heating treatment therefore induces a complete desorption of fluorine atoms for higher temperatures while only near a third of the initial amount of oxygen is thermally removed from the carbon surface. The atomic concentrations were calculated from XPS analysis, taking into account relative cross-sectional values: the points at RT in Figure 2 are relative to the as-functionalized structure ([C] = 63 at %, [F] = 24 at % and [O] = 13 at %), and the subsequent heating steps are connected by full lines. The atomic concentration of a pristine sample is added to the graph ([C] = 98 at %, [F] = 0 at %, [O] = 2 at %) as a reference. Upon heating, the fluorine-containing species desorb (red symbols), explaining the decrease in the F 1s core level intensity. At 540 °C, the fluorine concentration decreases to 0.5 at %, indicating that almost all fluorine atoms desorb from the carbon nanotube surface. This temperature is slightly higher with respect to *T* = 400 °C as reported for single-walled carbon nanotubes [38] but consistent with the thermal desorption range published by Gu et al. [39] and Bulusheva et al. [37]. It is important to notice that the remaining relative amount of oxygen bound to fluorinated carbon at 533.8 eV, is near 1 at % at *T* = 540 °C (Figure 1c), which is consistent with the residual total atomic concentration of fluorine. This vanishes completely at *T* = 900 °C once the total desorption of fluorine is confirmed by the complete disappearance of the F 1s signal.

From a close inspection of Figure 2, we can observe that while the fluorine concentration has a sharp decrease with increasing temperature, the oxygen concentration shows a more gradual trend. The small decrease in the concentration of the oxygen-containing species up to 430 °C was reported to be related to the desorption of physically adsorbed oxygen associated with the formation of hydroxyperoxide after air exposure of freshly functionalized nanotubes [14]. A recent study reported on the improved hydrophilicity of carbon nanostructures upon oxyfluorination [40]. Indeed the presence of hydroxylic groups on the surface attracts water adsorption, in contrast to the case of pure fluorinated carbon systems that show strong hydrophobicity [20]. In addition to the presence of oxygen atoms grafted during plasma treatment, we must consider that defects such as vacancies created during the process are preferential sites for adsorption. As soon as the sample is removed from the vacuum chamber, they may also contribute to C–O bonding, but it is not obvious to separate their contribution from that of carbon–oxygen bonds generated during plasma fluorination. A remaining 8% of oxygen-containing species do not desorb from the carbon structures because the single and double C–O bonds are stronger with respect to the multiple fluorinated carbon groups. Actually, binding energies of 2.37, 1.04 and 0.9 eV were theoretically calculated for fluorine directly bound to C, for CF_2_ and CF_3_, respectively [37]. The chemisorption of oxygen leads to binding energy values ranging from 3–5 eV, depending on the chemisorption site [41].

**Figure 3:**
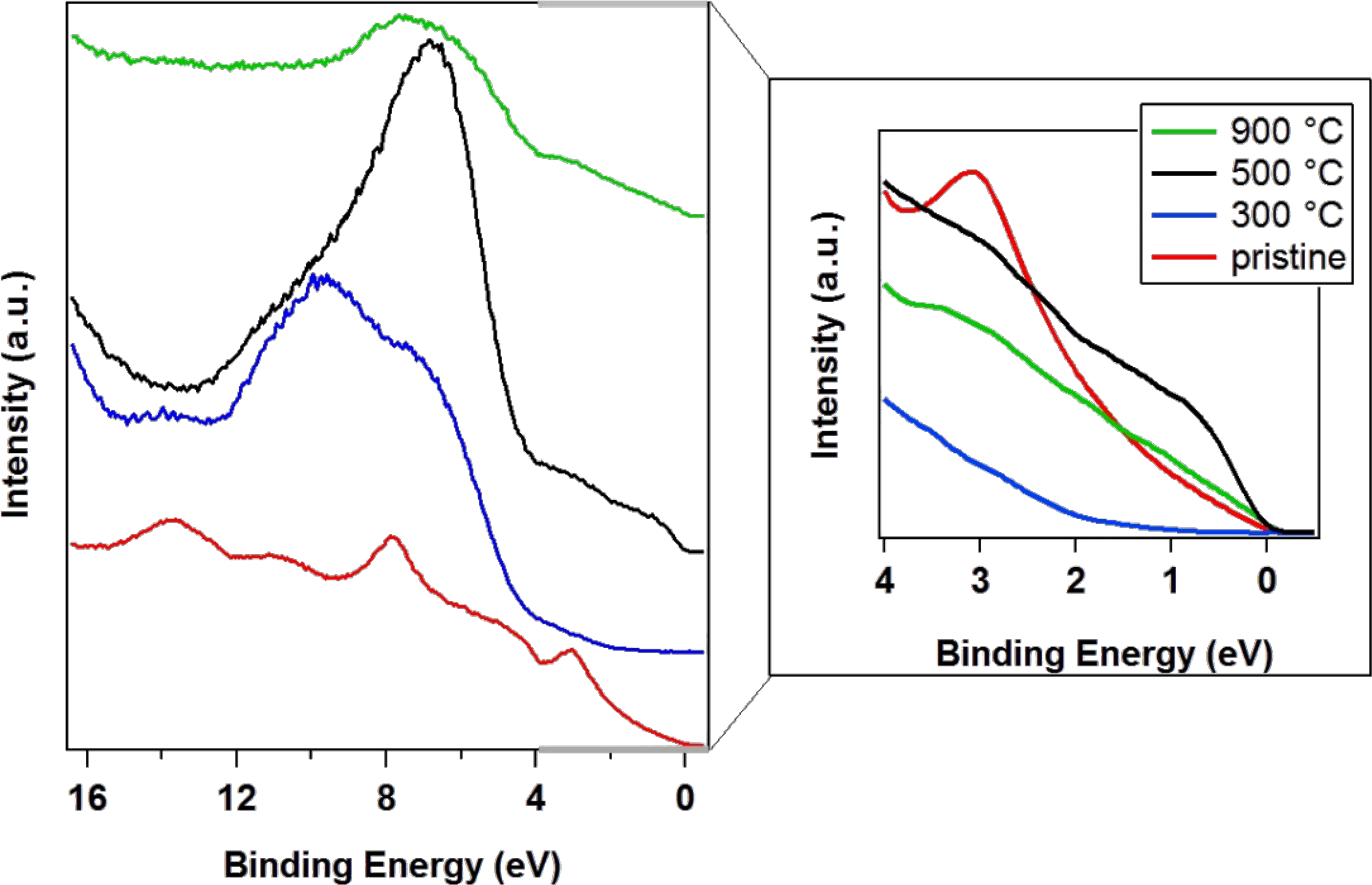
UPS spectra acquired with a photon energy of *h*ν = 31 eV, the red line is the pristine vCNT sample, the blue is the functionalized, annealed (*T* = 300 °C) sample, the black and the green lines are related to samples heated at 500 and 900 °C, respectively.

In order to understand the effects of fluorination in valence electronic states of the vCNTs, UPS measurements were performed (Figure 3). First, we analyzed a pristine sample (red curve) after prolonged heating in UHV for removing possible adsorbed contaminations. The features related to C–C bonds are clearly distinguishable: C–C π-states appear around 3 eV, C–C σ-states are around 8 eV, and σ–π hybridized states are localized at higher binding energy, from around 10 eV [6]. The functionalized sample annealed to *T* = 300 °C (blue curve) shows the dominating structures generated by the grafted species [42–43] due to their higher cross section for photoemission with respect to carbon (*h*ν = 31 eV): the O 2p-like states around 7 eV, the C–F bonding orbitals and the F 2p-like states around 9 and 11 eV, respectively. The strong modification in the intensity ratio of the structures in the UPS spectrum is due to the fluorine grafting that also drastically reduces the relative intensity associated with the density of states just below the Fermi level. This is a signature of a drastic change in the electronic properties with respect to the pristine metallic behavior.

Upon heating at 500 °C, the fluorine-related states appear at 11 eV as a low intensity shoulder with respect to the high intensity feature generated by oxygen-related states (black curve). Since the cross section for photoemission from the O 2p-like states and F 2p-like states are almost the same when using a photon energy of *h*ν = 31 eV, it can be suggested that, when in high amounts, the structure generated by fluorine atoms prevails over that of oxygen-related states in valence band spectra. Fluorine atoms mainly bind to carbon, generating an inductive effect on the overall electronic structure. While during the heating process, fluorine desorbs from the surface, oxygen atoms, which were participating in the plasma-generated C–O, stay bound. The oxygen-related states are no longer affected by the presence of fluorine and they are more intense. Moreover, the relative intensity related to the density of states near the Fermi level increases again. This shows that the oxygen effect in the electronic properties is less pronounced at this concentration. This is in contrast to the presence of fluorine, which induces a semiconducting/insulating effect. Indeed, F atoms attract electrons from the carbon lattice due to their higher electronegativity, thus reducing the charge in the conducting π-orbitals, introducing scattering centers and disrupting the carbon structure.

At the final heating step (*T* = 900 °C), the oxygen concentration is reduced, as well as the contribution related to C–O states in the valence band region (green curve). Low intensity structures related with C–C states can be resolved again, however, the pristine spectrum cannot be recovered. This result confirms the XPS study previously described. It is possible to finely tune the density of electronic states near the Fermi level by heat treatment in UHV [6]. However, if oxygen atoms are simultaneously grafted during the plasma fluorination, it is not possible to obtain a perfect defunctionalization due to the presence of strong C–O bonds.

**Figure 4:**
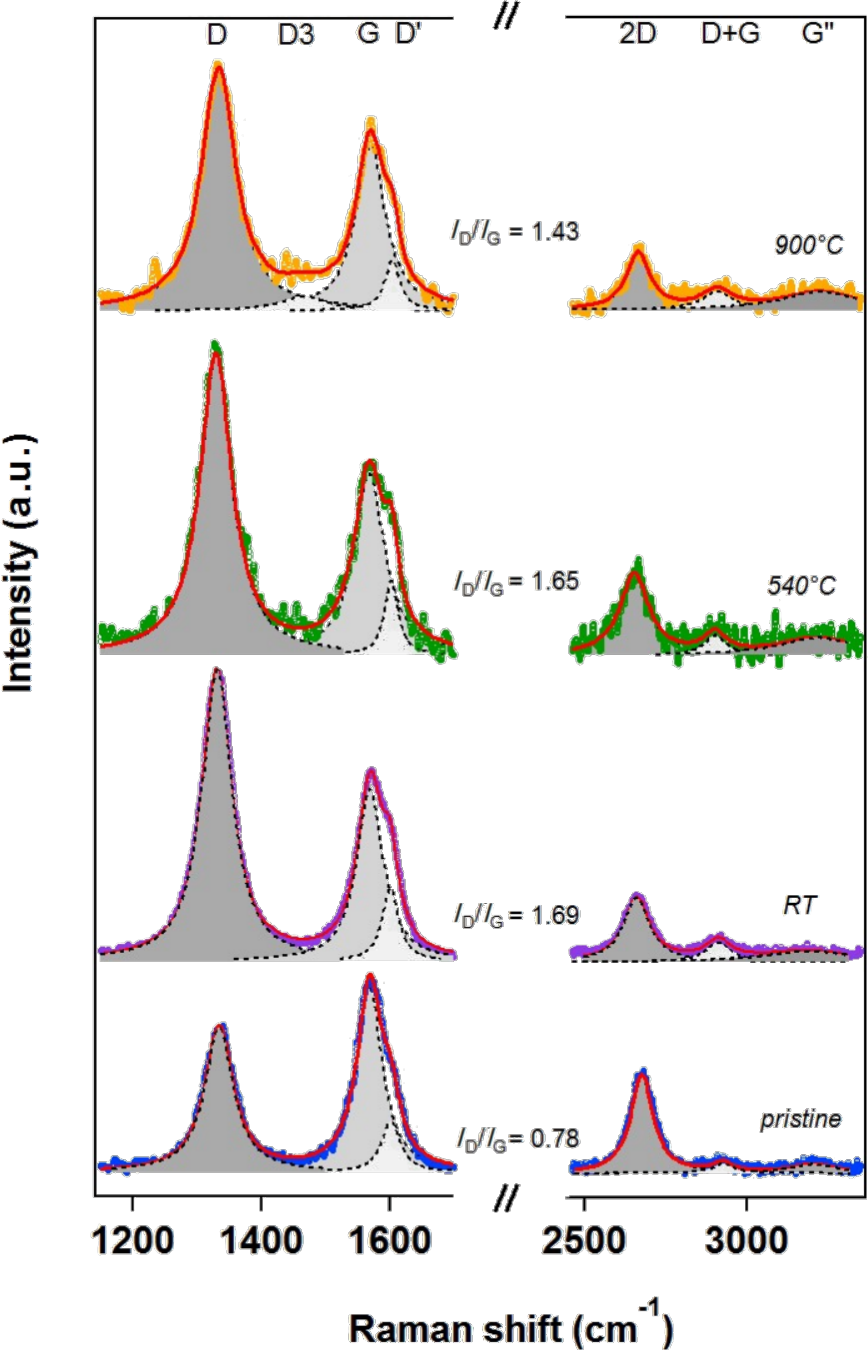
Raman spectra acquired on pristine, functionalized and thermally treated vCNTs. The spectrum at the top was recorded after heating the sample to *T* = 900 °C, which corresponds to a completely defluorinated sample. The spectra were normalized to the G-peak intensity.

The Raman spectroscopy results displayed in Figure 4 confirm the impact of the plasma fluorination on the vibrational modes of the pristine sample. This technique is often used for a qualitative investigation of the functionalization effect on carbon nanotubes structures [2,44–47] and to quantify the defect density in the CNTs sidewalls. However, a qualitatively analysis approach is more adequate in the present case.

The Raman spectra were collected from pristine, functionalized, heat-treated (*T* = 540 °C) and finally high-temperature annealed (*T* = 900 °C) sample (Figure 4). The most interesting features are in the first-order Raman spectra: the disorder-induced D-band (around 1335 cm^−1^), D’-band (around 1602 cm^−1^, attributed to intravalley scattering activated through a double resonance process), and the tangential G-mode (at 1570 cm^−1^). The last feature is related to the E_2g_ Raman-active mode, where the two atoms in the graphene unit cell vibrate tangentially against one another. The second-order Raman spectra is characterized by a 2D-band, which occurs around 2660 cm^−1^, the D+G band at 2900 cm^−1^ (as a result of the combination of D and G modes), and the G”-band at 3220 cm^−1^ (that can be explained as the first overtone of the D’-band) [48–49]. For the spectrum recorded after heating the sample to 900 °C, an additional D_3_-peak (1466 cm^−1^) was necessary for the fitting. The origin of this mode is not clear, but it has been attributed to amorphous carbon fraction of soot (e.g., organic molecules, fragments or functional groups) or to an approximate background evaluation [49–51].

The D-mode has been largely used as a diagnostic for disruptions in the hexagonal lattice of carbon nanotubes. The relative intensity of this mode can provide direct evidence of covalent modification and defect concentration. The D-band is associated to the A_1g_-mode breathing vibrations of six-membered sp^2^ carbon rings. It becomes Raman active after neighboring sp^2^ carbons are converted to sp^3^ hybridization in graphitic materials. This can be due to the presence of in-plane substitutional heteroatom vacancies, grain boundaries, or other symmetry-breaking defects or damage of the lattice [52]. In Figure 4, the pristine sample already shows a high intensity D-peak when compared to the plasma-fluorinated nanotubes. This is because of the sp^3^ behavior and intrinsic defects revealed particularly when acquiring the Raman spectrum from a region including the tips of vertically aligned carbon nanotubes. The information about the disorder and defect/damage induced by the functionalization is directly qualitatively inferred from the *I*_D_/*I*_G_ ratio: in the pristine sample (blue curve) this ratio is 0.78, and increases to 1.69 for the as-functionalized sample (violet curve); it changes to 1.65 upon heating at 540 °C (green curve), and decreases to 1.43 upon heating at *T* = 900 °C (orange curve). Moreover, the width of the D-band increased with functionalization (from 53.6 cm^−1^ to 56.6 cm^−1^) but also with the heating treatment (60.5 cm^−1^ and 64.5 cm^−1^). The remaining disorder mode therefore indicates both the enhancement of disorder in the carbon lattice upon functionalization, and the increase in the amount of defects. This has been associated with the local carbon removal on the sidewall during the heating-induced desorption process, as for example, via recombination of mobile species ending in formation of COF_2_ and CF_4_ at high temperature [37]. However, a partial rearrangement of the carbon lattice upon heating to higher temperature (900 °C) most probably takes place, as confirmed by the 13.3% reduction in the *I*_D_/*I*_G_ ratio as the carbon bonding at the walls was repaired. This is in agreement with the results about recovering of the sidewall upon hydrazine treatment or heating treatment for SWCNTs where the partial recovery of the bi-dimensional graphene lattice was demonstrated by the strong decrease in the intensity of the D-band [38,53]. A similar trend was obtained from peak area ratios, as it is often used in the case of a high degree of disorder [51]. In this regard, the following values were calculated from our spectra: *A*_D_/*A*_G_ = 0.88 (pristine), 1.92 (functionalized), 1.74 (functionalized and heated at 540 °C), 1.68 (functionalized and heated at 900 °C), confirming the evolution of *I*_D_/*I*_G_.

As a consequence of the Ar/F_2_ plasma functionalization, structural defects are generated with respect to the pristine sample, as also confirmed by the broadened base of the C 1s core level in Figure 1a and the *A*_D_/*A*_G_ ratio [18]. Through these defects, the functionalization took place, leading to amorphization of the carbon lattice. The thermal-heating-induced desorption of grafting species and a self-healing process was activated by the carbon lattice, although a complete removal of defective carbon was not possible due to the presence of C–O bonds. Despite the strong impact of the plasma functionalization, the intrinsic characteristics of vCNTs were mostly preserved: the nanotube structure was observed in TEM (not shown), and the characteristic sp^2^ component in the C 1s core level (as well as the G-band in the Raman spectrum) was still present, indicating that the electronic properties were retained.

## Conclusion

In the present work, we have described the plasma fluorination of vertically aligned carbon nanotubes using an Ar/F mixture precursor gas. The analysis of the impact of fluorination on the pristine nanotube structure was supported by Raman and photoemission spectroscopy. The exposure to a Ar/F_2_ (95:5) plasma resulted in a continuous creation of active sites through Ar ions. However, the selectivity towards fluorine species was lost with respect to contaminant oxygen present in the chamber as water vapor that was decomposed by the energetic ions during the plasma process. The grafted fluorine and oxygen atoms caused the hybridization change from sp^2^ to sp^3^ of carbon atoms. The evolution of the Raman D-band intensity showed an increase in the disorder due to the functionalization. This is presumably a consequence of tip and sidewall damage that partially disrupt the sp^2^ carbon lattice. A subsequent decrease in the D-band intensity was observed as the sidewall was partially repaired after the defluorination process initiated by heating at higher temperature. This is in agreement with the recovering of the sidewall upon hydrazine treatment. Hence, on one hand, the heating promoted desorption of fluorine-grafted species, while on the other, it induced the self-healing of the carbon lattice recovery. Despite the drastic changes observed as consequence of fluorination and heating treatment, the G-band and the sp^2^ component in C 1s were globally preserved. This suggests that the intrinsic properties of the vCNTs were not permanently altered. The thermal desorption was analyzed by photoemission spectroscopy, following the evolution of core levels and valence band states. While fluorine-grafted species desorbed almost completely at around 540 °C, the oxygen atoms were strongly bound to the carbon lattice through single and double C–O covalent bonds. This illustrated its electronic states also in the valence band, where we observed a fine tuning of the density of states near the Fermi level by the desorption of fluorine.

## Supporting Information

File 1 Additional experimental information.
Fitting procedure of C 1s core level and the table of XPS analysis results are available for the as-functionalized sample.
