# Supplementary material for: Plasma fluorination of vertically aligned carbon nanotubes: functionalization and thermal stability
Source: Beilstein J Nanotechnol. 2015 Dec 1;6:2263–71. doi: 10.3762/bjnano.6.232 (PMC4685895; doi:10.3762/bjnano.6.232)
Supplement: File 1 — Additional experimental information. Fitting procedure of C 1s core level and the table of XPS analysis results are available for the as-functionalized sample. [file Beilstein_J_Nanotechnol-06-2263-s001.pdf]

Supporting Information

for

Plasma fluorination of vertically aligned carbon nanotubes: functionalization and thermal stability

Claudia Struzzi[1], Mattia Scardamaglia[1], Axel Hemberg[2], Luca Petaccia[3], Jean-François Colomer[4], Rony Snyders[1,2] and Carla Bittencourt[1*§]

Address: [1]Chimie des Interactions Plasma-Surface, CIRMAP, University of Mons, 7000 Mons, Belgium, [2]Materia Nova Research Center, 7000 Mons, Belgium, [3]Elettra Sincrotrone Trieste, Strada Statale 14 km 163.5, 34149 Trieste, Italy, and [4]Research Group on Carbon Nanostructures (CARBONNAGe), University of Namur, 5000 Namur, Belgium

Email: Carla Bittencourt* - carla.bittencourt@umons.ac.be

*Corresponding author

[§]Tel.: +32 065554963

Additional experimental information.

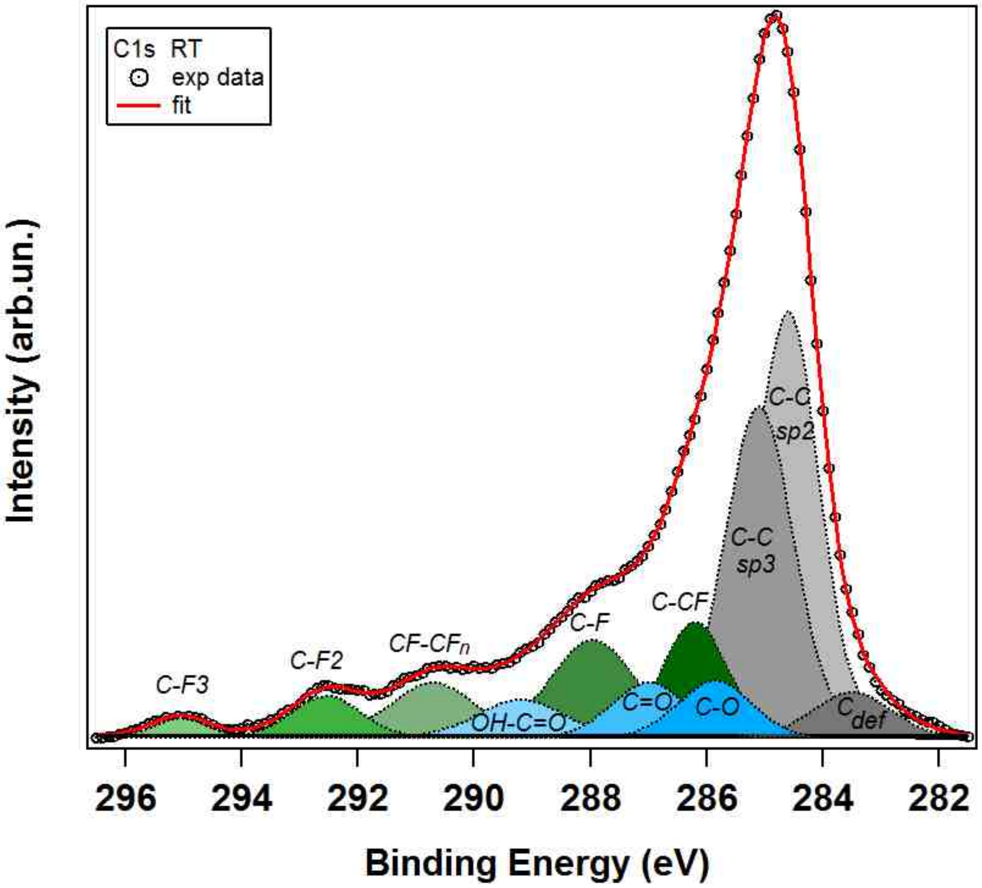

Figure S1: XPS curve fitting of as-functionalized vCNT referred to room temperature (RT) case in the article. The C_{1s} experimental data recorded after the fluorination are represented by black markers, the curve fitting is the red line over imposed. The grey scale colored curves are fitting components related to carbon-carbon bond (C_{def} usually associated to ion induced defective carbon, C-C bonds in sp^2 and sp^3 configurations), the blue ones are related to carbon bound to oxygen (C-O, C=O, OH-C=O), while carbon bound to fluorine atoms are reported as green scale curves (C-CF, C-F, CF-CF_n, C-F_2 and C-F_3).

	F1s				O1s			
**RT**	9.0%	32.3%	33.8%	24.9%	12.4%	25.2%	52.1%	10.3%
**BE (eV)**	688.7	687.4	685.8	684.3	533.8	532.5	531.0	529.8
**FWHM (eV)**	1.8	2.1	1.9	2.1	2.0	1.6	1.6	1.8
**150 °C**	9.5%	47.6%	36.3%	6.6%	12.7%	30.5%	55.4%	1.4%
**BE (eV)**	688.7	687.3	686.0	684.4	533.7	532.4	531.2	529.2
**FWHM (eV)**	1.6	2.0	2.1	1.7	1.9	1.7	1.5	1.3
**300 °C**	9.4%	75.7%	14.9%	-	13.0%	18.8%	68.2%	-
**BE (eV)**	688.7	687.3	685.7	-	533.9	532.7	531.5	-
**FWHM (eV)**	2.0	1.9	2.1	-	1.7	1.4	1.5	-
**430 °C**	5.5%	83.8%	10.7%	-	8.6%	17.0%	74.4%	-
**BE (eV)**	688.7	687.4	685.7	-	534.1	532.7	531.7	-
**FWHM (eV)**	2.4	1.9	1.8	-	1.5	1.4	1.6	-
**540 °C**	12.6%	59.0%	28.4%	-	5.6%	16.6%	77.8%	-
**BE (eV)**	688.8	687.3	685.8	-	534.1	532.8	531.8	-
**FWHM (eV)**	2.3	2.1	2.0	-	1.2	1.3	1.5	-
**900 °C**	-	-	-	-	-	23.1%	76.9%	-
**BE (eV)**	-	-	-	-	-	532.7	531.4	-
**FWHM (eV)**	-	-	-	-	-	1.7	1.8	-

Table S1: Summary of the peak fitting analysis of F_{1s} and O_{1s} core level spectra recorded from functionalized sample (RT) and for all heating steps (150 °C, 300 °C, 430 °C, 540 °C, 900 °C). Relative area values (%), position of the components (BE, binding energy) and full width at half maximum (FWHM) are listed.
